# The Use of Common Warts of the Hand in Skin Grafting

**Published:** 1878-09

**Authors:** 


The Use of Common Warts of the Hand in Skin-Grafting.—As common warts of the skin are collections of vascular papillæ, admitting of easy separation without injury to their excessively thick layer of well-nourished epidermis, the idea was conceived that, by their use for the purpose of skin-grafting, better and more rapid results would be obtained than when the ordinary skin of less vitality is used. As proof of the theory, the following case is cited, where there had complete destruction of all the skin on the dorsum of the foot, involving to a great extent the deep cellular tissue, and where for several weeks no healing advanced until grafts of freshly removed warts from the patient's hand immediately started little islands of tissue, which rapidly increased until they coalesced and met the margins of the border skin, thereby completely covering the foot by firm, protecting integument.

Samuel Root, aged twenty years, by occupation an iron moulder, while at work had a stream of melted iron run over his foot and through an opening in the front of his boot—a portion being moulded between a thick sock, which was burned to a black powder, and the remains of his boot. In about a minute his foot was placed in a pail of cold water, and as the boot and sock were hastily removed, a large mass of adherent skin was torn off from the crown of his foot. Extensive gangrene and sloughing of tissue followed, but treatment, in four weeks, left a healthy-looking granulating wound. Although strapping, hermetically sealing, removal of pressure, retention of foot while in bed in an elevated position, with applications of the usual healing balsams, and submersion in hot water were resorted to, yet, for the next five weeks, not the slightest advance towards healing occurred; the skin at the margin of the wound being exceedingly thin and shiny, while the wound itself was a mass of exuberant granulating tissue. For a number of days epithelial scrapings were dusted on the wound without any apparent effect. At the end of nine weeks I removed two large warts from his hand by transverse incisions through their proximal parts, carefully wiped off all blood and tore each wart into quarters, in the direction of its papillæ, and these eight portions of warts were planted about equal distances from each other through little cuts, and left deposited about a line beneath the surface, all being completely covered at the end of the operation by the overlapping granulating tissue. The entire wound was now hermetically sealed by a covering of mild balsam of Peru ointment spread on soft lint, retained in position by adhesive straps, which were not at any time permitted to come in direct contact with the sore. At the end of forty-eight hours he walked on crutches over half a mile to my office. The dressings, in consequence of the purulent secretions, were easily removed. Each of the little grafts had received nourishishment, and all were alive; nor did any of them slough, as we nearly always see before the white zone shows itself in the ordinary skin grafting, but without any degeneration continued to grow, forming new skin, until they joined together, and at the end of three weeks from the time of grafting, and twelve weeks from the time of the original burn, the foot was entirely covered by skin and firm cicatricial tissue. The cicatrix then, on November 17, 1874, measured three by four and a half inches. Three years afterward, on examination, it was nearly as large, but the cure remained complete.

Warts of the hand can be used with better results than small pieces of normal skin, in skin-grafting, in consequence of being easily separated uninjured into numerous cylindrical rods of great vascularity and containing a large proportion of hypertrophied epithelium, which, when planted in healthy granulating tissue, readily adapt themselves to the new soil, receiving direct nourishment and quickly growing as starting-points for a new and smooth epithelial covering.—*The Medical Record.*

